# Mild early‐life stress exaggerates the impact of acute stress on corticolimbic resting‐state functional connectivity

**DOI:** 10.1111/ejn.15538

**Published:** 2021-12-01

**Authors:** Huan Wang, Judith M. C. van Leeuwen, Lycia D. de Voogd, Robbert‐Jan Verkes, Benno Roozendaal, Guillén Fernández, Erno J. Hermans

**Affiliations:** ^1^ Donders Institute for Brain, Cognition and Behaviour Radboud University Medical Center, Radboud University Nijmegen The Netherlands

**Keywords:** cortisol, early‐life stress, functional connectivity, HPA axis, stress response

## Abstract

Abundant evidence shows that early‐life stress (ELS) predisposes for the development of stress‐related psychopathology when exposed to stressors later in life, but the underlying mechanisms remain unclear. To study predisposing effects of mild ELS on stress sensitivity, we examined in a healthy human population the impact of a history of ELS on acute stress‐related changes in corticolimbic circuits involved in emotional processing (i.e., amygdala, hippocampus and ventromedial prefrontal cortex [vmPFC]). Healthy young male participants (*n* = 120) underwent resting‐state functional magnetic resonance imaging (fMRI) in two separate sessions (stress induction vs. control). The Childhood Trauma Questionnaire (CTQ) was administered to index self‐reported ELS, and stress induction was verified using salivary cortisol, blood pressure, heart rate and subjective affect. Our findings show that self‐reported ELS was negatively associated with baseline cortisol, but not with the acute stress‐induced cortisol response. Critically, individuals with more self‐reported ELS exhibited an exaggerated reduction of functional connectivity in corticolimbic circuits under acute stress. A mediation analysis showed that the association between ELS and stress‐induced changes in amygdala–hippocampal connectivity became stronger when controlling for basal cortisol. Our findings show, in a healthy sample, that the effects of mild ELS on functioning of corticolimbic circuits only become apparent when exposed to an acute stressor and may be buffered by adaptations in hypothalamic–pituitary–adrenal axis function. Overall, our findings might reveal a potential mechanism whereby even mild ELS might confer vulnerability to exposure to stressors later in adulthood.

## Graphical abstract

Childhood trauma has long‐lasting consequences for stress coping and confers vulnerability for development of stress‐related disorders. Here, we show that a history of mild early‐life stress in an otherwise healthy male population exacerbates the effects of an acute laboratory stressor on functioning of neural circuits involved in emotional regulation (amygdala, hippocampus and vmPFC), thereby revealing a mechanism by which even mild early‐life stress can enhance vulnerability to stressors.

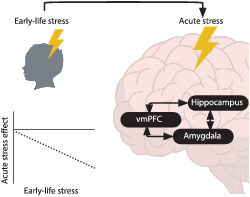

**Abbreviations**
AA: alpha‐amylase
ACTH: adrenocorticotropic hormone
ANCOVA: analysis of covariance
BDI: Beck Depression Inventory
BOLD: blood‐oxygen‐level‐depend
BP: blood pressure
CTQ: Childhood Trauma Questionnaire
dlPFC: dorsolateral PFC
DMN: default‐mode network
DTI: diffusion tensor imaging
ELS: early‐life stress
EPI: echo‐planar imaging
FDR: false‐discovery rate
fMRI: functional magnetic resonance imaging
GR: glucocorticoid receptor
HPA: hypothalamic–pituitary–adrenal
HRF: heart‐rate frequency
HRV: heart‐rate variability
MPRAGE: magnetization‐prepared rapid gradient echo
MR: mineralocorticoid receptor
NEO‐FFI: NEO Five‐Factor Inventory
PFC: prefrontal cortex
PTSD: post‐traumatic stress disorder
RETROICOR: retrospective image‐based correction
ROIs: regions of interest
SAM: sympathetic adrenomedullary
STAI: State/Trait Anxiety Inventory
vlPFC: ventrolateral PFC
vmPFC: ventromedial PFC

## 1 INTRODUCTION

A history of prolonged stress or trauma (e.g., neglect or abuse) during childhood is a known vulnerability factor for the development of various forms of psychopathology in response to stressful life events later in life (Teicher et al., 2003). Research into lasting effects of early‐life stress (ELS) have revealed changes in brain structure and function (Teicher et al., 2016) but also alterations in neuroendocrine stress‐response systems such as the hypothalamic–pituitary–adrenal (HPA) axis (Koss & Gunnar, 2018; Tarullo & Gunnar, 2006; van Bodegom et al., 2017). Together, these findings suggest that the effects of acute stress on brain function may be altered by a history of ELS. Long‐term effects of milder forms of ELS may furthermore only manifest themselves in states of acute stress.

Acute stress affects brain function through two interrelated systems: the sympathetic adrenomedullary (SAM) (Frankenhaeuser, 1986) and the HPA (Stratakis & Chrousos, 1995) systems. The SAM system orchestrates the fight/flight response (Cannon, 1953) and is accompanied by the rapid central release of catecholamines such as norepinephrine. At a slower pace, the HPA axis triggers release of glucocorticoids, which can act synergistically with catecholamines through non‐genomic mechanisms (Groeneweg et al., 2011; Roozendaal et al., 2006), but also exerts antagonistic effects by altering genomic transcription (Datson et al., 2001). Notably, (beta‐)adrenergic, mineralocorticoid (MR) and glucocorticoid (GR) receptors are highly expressed in corticolimbic circuits, including the amygdala, hippocampus and prefrontal cortex (PFC) (Arnsten & Li, 2005; Joëls et al., 2012; Lupien et al., 2007). Amygdala–PFC circuits are implicated in cognitive control of emotion (Hartley & Phelps, 2010; Ochsner et al., 2002; Ochsner & Gross, 2008; Sotres‐Bayon et al., 2006), amygdala–hippocampal coupling supports contextualization and emotional modulation of memory (Fanselow, 2000; Gerraty et al., 2014; Zeithamova et al., 2012), whereas hippocampal projections to the PFC are involved in incorporating novel information into long‐term memory (Gerraty et al., 2014; Van Kesteren et al., 2010; Zeithamova et al., 2012). Thus, stress‐related hormones and neurotransmitters have a major influence on the neural circuits that are critical in regulation of affective processes.

Research combining human neuroimaging with controlled manipulation of acute stress has shown that resting‐state functional connectivity within these circuits is indeed altered following controlled induction of acute stress. For instance, resting‐state (i.e., in absence of an explicit task) functional connectivity between amygdala and hippocampus was enhanced immediately after acute stress, and this increase persisted for multiple hours (Vaisvaser et al., 2013). Another study showed that resting‐state functional connectivity between amygdala and ventromedial PFC (vmPFC) was increased 60 min after stress induction (Veer et al., 2011). Effects of acute stress on resting‐state functional connectivity within corticolimbic circuits have furthermore shown to be timing dependent: Immediately after stress induction, resting‐state functional connectivity between the amygdala and parahippocampal gyrus was increased, and coupling with ventrolateral PFC (vlPFC) was decreased. More than 30 min after stress (when cortisol levels peak or start returning to baseline), by contrast, coupling with dorsolateral PFC (dlPFC) was decreased, while cortisol responder and non‐responder groups had diverging patterns of amygdala–PFC connectivity to acute stress (Quaedflieg et al., 2015). Thus, effects of acute stress on subsequent resting‐state functional connectivity within circuits affected by stress‐related hormones and neurotransmitters show a dynamic pattern of transient changes in coupling between amygdala, hippocampus and PFC.

More lasting changes have been observed in individuals who have a history of exposure to ELS. For instance, ELS is associated with cardiovascular (Suglia et al., 2018), metabolic (Danese & Tan, 2014) and immune system (Coelho et al., 2014) dysfunction, and it is a known risk factor for development of psychiatric conditions such as post‐traumatic stress disorder (PTSD), major depression and anxiety disorders (Chapman et al., 2004; Famularo et al., 1992; Felitti et al., 2019; Hicks et al., 2009; Kendler et al., 2003; McCauley et al., 1997; Pelcovitz et al., 1994). One potential underlying mechanism of such ELS effects is a prolonged alteration of functioning of stress‐response systems (Gunnar & Quevedo, 2007). For instance, ELS causes a change in the ability of circulating glucocorticoids to exert negative feedback on activation of the HPA axis (Baes et al., 2012; Heim et al., 2008; Nemeroff et al., 2003), most likely through epigenetic mechanisms. ELS is associated with lower adrenocorticotropic hormone (ACTH) (Carpenter et al., 2007), lower baseline cortisol and blunted cortisol responses to psychosocial stressors (Carpenter et al., 2009; Carpenter et al., 2011), which further interact with genotype (Heim & Binder, 2012) and thereby contribute to developmental programming of neuroendocrine and behavioural responses to stress (van Bodegom et al., 2017).

Human neuroimaging studies have furthermore shown a wealth of effects of ELS on brain structure and function in adulthood. One consistent finding is that ELS is associated with reduced volume of hippocampus, amygdala and PFC (Teicher et al., 2016). Functional connectivity between these regions was similarly shown to be affected (Teicher et al., 2016). For instance, ELS negatively predicts resting‐state functional connectivity between hippocampus and vmPFC (Herringa et al., 2013), as well as resting‐state functional connectivity between amygdala and an insular‐hippocampal region (van der Werff et al., 2013). Thus, effects of acute stress and ELS on neural function intersect in core neural circuits supporting affect regulation. This suggests that, potentially through mechanisms involving altered functioning of the HPA axis, effects of acute stress on subsequent neural function may be exacerbated by a history of ELS. Such interactive effects may play a key role in conferring vulnerability in healthy people with a history of ELS but have not been investigated.

We therefore examined, in a population of 120 adult healthy male volunteers, how a history of mild ELS alters the effects of experimentally induced acute stress on subsequent resting‐state functional connectivity between amygdala, hippocampus and vmPFC. In line with earlier work on effects of ELS and acute stress, we examined functional connectivity at rest, in the aftermath of exposure to an acute stressor as a proxy for functioning of these corticolimbic circuits. We focused on the ventromedial part of PFC as this region is recognized as a pathway in which prefrontal affect regulatory circuits converge (Delgado et al., 2008; Diekhof et al., 2011). Critically, we examined a healthy population with levels of self‐reported ELS ranging from no to mild ELS. Our reasoning behind this was that (1) we aimed to disentangle predisposing effects of mild ELS from consequences of development of psychopathology, which would be confounded in patients with a history of ELS (Admon et al., 2013), and (2) it remains largely elusive whether mild ELS in otherwise healthy subjects has long‐lasting consequences for how acute stress affects brain function. Our design enabled us to (1) determine effects of ELS on basal cortisol; (2) examine effects of ELS on stress‐induced mood and physiology; (3) critically test the interaction between ELS and acute stress on resting‐state functional connectivity between the amygdala, hippocampus and vmPFC; and (4) examine the role of the potential alterations in HPA‐axis function in this interaction. Here, we follow earlier work indicating that lower cortisol baseline levels may reflect increased GR sensitivity (Alexander et al., 2018; Heim et al., 2008; Heim & Binder, 2012), which in turn may play a role in mediating ELS effects on stress‐induced changes in resting‐state brain function.

## 2 MATERIAL AND METHODS

### 2.1 Participants

One‐hundred‐and‐twenty healthy right‐handed male participants, between 18 and 30 years of age, were recruited for this study (see Table 1 for demographics and questionnaire scores). The reason we only recruited male participants was to avoid the known variation in stress responses caused by menstrual cycle and/or use of hormonal contraception (Kirschbaum et al., 1999). Exclusion criteria were (1) current or history of any psychiatric, neurological or endocrine disorder (assessed using self‐report); (2) regular use of psychoactive drugs during the last 6 months; (3) habitual smoking or regular use of recreational drugs; (4) magnetic resonance imaging (MRI) contraindications; (5) irregular sleep or intense daily exercise; (6) hearing or (uncorrected) vision restrictions; and (7) regular use of corticosteroids. Five participants were excluded from analyses: One was excluded due to movement‐related problems in segmenting the T1‐weighted scan, two were excluded because of falling asleep during testing and two were excluded because of excessive movement (>4 SD above the mean voxel‐wise displacement) during one of the resting‐state scans. All participants granted written informed consent and were paid for participation (€60). This study was approved by the local ethical review board (CMO region Arnhem‐Nijmegen, the Netherlands).

**TABLE 1:** Characteristics of the study population (*n* = 115)

	Mean	SD	Range	Spearman's correlations with CTQ score
CTQ scores^a^	32.8	6.3	25–56	‐
Age	22	2.6	18.1–30.8	.130
BMI	22.6	2.3	17.3–30	.017
NEO‐FFI scores^a^				
Neuroticism^a^	28.4	6.7	14–50	.289**
Extraversion^a^	42.4	6.0	23–53	−.362**
Openness^a^	40.5	6.1	28–57	−.075
Agreeableness^a^	40.8	4.2	26–51	−.210*
Conscientiousness^a^	40.9	5.2	25–52	−.097
STAI‐t score^a^	35.5	7.7	21–60	.383**
BDI score^a^	4.3	4.1	0–18	.260**
Interval between sessions (days)	12.7	13.0	5–100	‐

Abbreviations: BDI, Beck Depression Inventory; BMI, body mass index; CTQ, Childhood Trauma Questionnaire; NEO‐FFI, NEO Five‐Factor Inventory; STAI‐t, State/Trait Anxiety Inventory (trait form).

^a^ These scores are within the normal range for a healthy, male population.

* *p* < .05.

** *p* < .01.

### 2.2 Design and procedure

**FIGURE 1:**
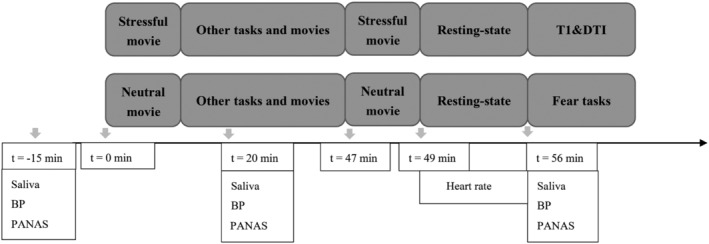
Overview of the study design. Every participant went through two sessions with a counterbalanced order: A stress session in which stressful movie clips were played several times and a neutral session in which neutral movie clips were played at the same time points as the stressful movie clips in stress session. The onset of the first movie clip was defined as *t* = 0 min, and this movie clip lasted 10 min. The resting‐state scan lasted 6.5 min. Different measures were obtained to verify stress responses at three different time points: Before the session at *t* = −15 min, after the first task at *t* = 20 min and before the last task at *t* = 56 min. Heart rate was recorded throughout resting‐state scanning. BP, blood pressure; DTI, diffusion tensor imaging; PANAS, Positive and Negative Affect Schedule

We analysed resting‐state functional MRI (fMRI) data that were acquired as part of a larger study on individual differences in the effects of stress on cognition (de Voogd et al., 2017; Everaerd et al., 2015; Henckens et al., 2015) (see Figure 1). In a within‐subjects design, participants underwent both a stress induction and neutral control session; the session order was counterbalanced. Stress was induced experimentally using four highly aversive movie clips that were played at different time points throughout the procedure. The first clip had a 10‐min duration (the onset of the first movie clip was defined as *t* = 0 min; see Figure 1), whereas the second to fourth clips served as reminders/boosters (each approximately 2‐min duration).

Highly aversive movie clips consisted of physically and sexually violent scenes taken from a commercial movie (Irréversible, 2002, by Gaspar Noé; Hermans et al., 2011). Neutral, non‐arousing scenes of another movie (Comment j'ai tué mon père, 2001, by Anne Fontaine) were shown during the neutral session at the same time points as during the stress session. The movie clips in stressful and neutral sessions were similar in the amount of speech, human (face) presence, luminance, environment and language.

The fourth movie clip (*t* = 47 min, relative to onset of the first movie clip) was followed by a resting‐state scan (6.5 min, *t* = 49 min). During resting‐state scans in both sessions, participants were instructed to keep their eyes closed but to remain alert and awake. Other experimental tasks, which have been reported elsewhere, alternated with movie clips before the resting‐state scan in each session. These tasks included a dynamic facial expression perception task (Everaerd et al., 2015; Henckens et al., 2015) (*t* = 10 min), an emotional conflict task (*t* = 23 min) (Kohn et al., 2017) and a face‐identity association task (Berkers et al., 2016; de Voogd et al., 2017) (*t* = 30 min). After the resting‐state scan, participants underwent a T1‐weighted and a diffusion tensor imaging (DTI) scan (in the stress session) or a fear conditioning task (Klumpers et al., 2015) (in the neutral session). The total duration of each scan session was approximately 105 min. The session order was counterbalanced and sessions were separated by an average of 2 weeks (minimally 5 days). All test sessions took place between noon and 8 PM to reduce diurnal variation in cortisol levels. For each participant, both sessions took place at the same time of day.

### 2.3 Childhood Trauma Questionnaire

Childhood Trauma Questionnaire (CTQ; Bernstein et al., 1997), our measure of ELS, was administered before the experimental sessions to assess experiences of ELS. CTQ includes five subscales: emotional abuse, emotional neglect, physical abuse, physical neglect and sexual abuse. Each subscale consisted of five questions, each with five answer options. In addition, Dutch versions of the State/Trait Anxiety Inventory (STAI) (Spielberger et al., 1970), the Beck Depression Inventory (BDI) (Beck et al., 1961) and the NEO Five‐Factor Inventory (NEO‐FFI) (McCrae & Costa, 2008) were completed by each participant.

### 2.4 Stress measurements

To validate the acute stress induction procedure, salivary cortisol and alpha‐amylase (AA), blood pressure (BP) and mood state were measured at three time points throughout the experiment. The first assessment was carried out before the start of the scanning procedure (*t* = −15 min, relative to onset of the first movie clip), a second following the first task (*t* = 20 min) and the final one at the end of the session (*t* = 56 min). Measures taken at the second time point (*t* = 20 min), for the stress condition in comparison with the neutral control condition, were regarded as an index of the stress response.

In addition to the three saliva samples taken during each experimental session, participants were asked to collect two extra samples at home during the day before the visit for the second session. The reason for this was to obtain a baseline cortisol measure unaffected by anticipation of a potentially stressful laboratory procedure. Participants were instructed to collect samples just prior to lunch (early afternoon) and just prior to dinner (late afternoon). The reason that these time points were chosen is that cortisol levels are relatively stable at these time points (Henckens et al., 2009; Henckens et al., 2010; Hermans et al., 2011; Qin et al., 2009) under non‐stressful conditions. To minimize variance in baseline cortisol levels, participants were instructed not to use any recreational drugs for at least 72 h prior to participation and to refrain from drinking alcohol, intense physical exercise and smoking for 24 h before each measurement. In addition to that, participants were instructed not to brush their teeth, floss or eat and drink anything but water for 2 h before each session, enabling adequate saliva sampling for cortisol assessment.

Salivette cotton swabs (Sarstedt, Rommelsdorf, Germany), which were placed in the participant's mouth, were used to obtain saliva samples. Participants were instructed to chew gently for 1 min to produce saliva. All samples were stored at −20°C until assaying. Laboratory analyses were performed at the Department of Biopsychology, Technical University of Dresden (Dresden, Germany). Biochemical analysis of free cortisol and AA in saliva was performed using a commercially available chemiluminescence immunoassay (IBL Inc.).

BP measurements were obtained using a standard automatic BP device and during the experiment in the MRI scanner using an AmbuloTM 2400 device. Mood state was measured by using the Positive and Negative Affect Schedule (PANAS) questionnaire (Watson et al., 1988).

A 50‐Hz pulse oximeter was used to measure heart rate during scanning. Raw pulse was processed offline using in‐house software for interactive visual artefact correction and peak detection and was used to specify fifth‐order Fourier models of the cardiac phase‐related modulation of the blood‐oxygen‐level‐depend (BOLD) signal (using retrospective image‐based correction [RETROICOR]; van Buuren et al., 2009), yielding 10 nuisance regressors that were included in multiple regression models used for fMRI analysis. Heart‐rate frequency (HRF) and variability (HRV; calculated as the root mean square of successive differences [rMSSD], an index of respiratory sinus arrhythmia) were also calculated offline. HRF and HRV time courses during the resting‐state scan were included as two additional nuisance regressors in fMRI analyses. Averaged HRF and HRV were used to test for stress‐induced differences between the two experimental sessions.

### 2.5 MRI data acquisition

Structural and functional MRI data were acquired on a Siemens (Erlangen, Germany) 1.5‐T Avanto MR scanner. In each session, we obtained 265 whole‐brain T2*‐weighted BOLD images using gradient echo‐planar imaging (EPI) with ascending slice acquisition and following parameters: 27 axial slices, 3.5‐mm slice thickness, repetition time (TR) = 1.49 s, echo time (TE) = 35 ms, flip angle = 80°; slice matrix = 64 × 64, slice gap = .7 mm, field of view (FOV) = 224 × 224 mm, bandwidth = 1906 Hz/px, echo spacing = .59 ms. The first five volumes were discarded to allow for T1 equilibration. A T1‐weighted image was acquired using a 3D magnetization‐prepared rapid gradient echo (MPRAGE) sequence with following parameters: TR = 2730 ms, TE = 2.95 ms, FOV = 176 × 256 × 256, voxel size: 1 mm isotropic.

### 2.6 MRI data preprocessing

All resting‐state EPI images were realigned and co‐registered to anatomical scans in native space to optimally accommodate interindividual structural variability of the regions of interest (ROIs). The bilateral hippocampus and amygdala were individually defined in native space using automated anatomical segmentation of T1‐weighted images using FSL FIRST (see http://fsl.fmrib.ox.ac.uk/fsl/fslwiki/FIRST). VmPFC was defined as a coordinate‐based sphere [*x* = 0, *y* = 40, *z* = −18, MNI152 reference space] with 10‐mm radius, which was registered into native space for each participant using reverse normalization. The coordinate was selected based on a meta‐analysis in which at this coordinate a peak was observed in the intersection of task‐related activation in the domains of fear extinction, placebo treatment and cognitive emotion regulation (Diekhof et al., 2011).

### 2.7 Time‐course extraction and calculation of functional connectivity

Resting‐state functional connectivity estimates were calculated for each pair of ROIs and for each session separately. First, we extracted and averaged the time courses of all voxels within the bilateral hippocampus, bilateral amygdala and the (medially located) vmPFC sphere, resulting in five time courses (two for amygdala, two for hippocampus and one for vmPFC). Before calculating functional connectivity, we applied a multiple regression model and residualization to remove nuisance signals from each time course. This model included six motion parameter regressors obtained from realignment (three translations and three rotations), the zero‐centred squares of the six motion parameters, the first derivatives of the six motion parameters, the zero‐centred squares of the derivatives of the six motion parameters for scan‐to‐scan motion (Chang et al., 2015; Woo et al., 2015), 10 RETROICOR cardiac phase regressors (Glover et al., 2000), an HRF regressor, an HRV regressor and a discrete cosine transform high‐pass filter with 1/128‐Hz cut‐off. For 10 participants, heart‐rate recording failed during scanning; therefore, these participants had 12 less nuisance regressors. Next, Pearson's correlations were computed for each pair of time courses, and these were Fisher's *z* transformed to transform these variables into normal distributions. After verifying that there were no hemispheric differences in connectivity measures (as well as their modulation by acute stress and ELS), we averaged all connectivity measures across hemispheres, thus resulting in three resting‐state functional connectivity parameters for each participant.

### 2.8 Statistical analyses

All statistical analyses were carried out in SPSS (Version 25). Effect size estimates are reported as partial eta squared (P*η* ^2^) for all relevant tests. Alpha was set at .05 throughout the tests. False‐discovery rate (FDR) correction was applied for each multiple test of same effect using the following formula: $Q*\left(i/m\right)$, where the outcome needs to be larger than the original *p*‐value in order for that test to survive multiple comparisons correction (Benjamini & Hochberg, 1995). *Q* stands for FDR (which was set at .05), *i* stands for the rank of the *p*‐value among all *p*‐values of same effect and *m* stands for the number of tests of same effect.

One‐sample Kolmogorov–Smirnov tests indicated that total CTQ scores were positively skewed, *D* (115) = .217, *p* < .001. Because this violates the assumptions of analyses of covariance (ANCOVAs), any main effect or interaction involving total CTQ scores was further verified using a robust, non‐parametric bootstrapping procedure. For each regression involving CTQ scores, we report bootstrapped (5000 iterations) parameter estimates (*β*) and 95% confidence intervals (CIs). Statistical significance is indicated when the 95% CI does not cross zero. To correct for multiple comparisons, we applied FDR correction to corresponding *p*‐values from the bootstrapped linear regression.

#### 2.8.1 Physiological and psychological outcome measures

To test the effects of ELS on basal HPA‐axis function, Spearman's rank order correlations were computed between total CTQ scores and basal cortisol. For completeness, we also report bootstrapped regression parameter estimates (5000 iterations) and 95% CIs for these. To confirm successful stress induction and investigate the association with ELS, we performed separate repeated‐measures ANCOVAs on the physiological and psychological measures at time point 2 (*t* = 20 min) with stress as within‐subject factor, session order as between‐subject factor and total CTQ score as covariate. We additionally ran a repeated‐measures ANCOVA to investigate the time by stress interaction on cortisol levels exclusively (see supporting information).

#### 2.8.2 Functional connectivity

To investigate the effect of stress on resting‐state functional connectivity between our three ROIs (amygdala–hippocampus, amygdala–vmPFC and vmPFC–hippocampus) and its association with ELS, we conducted three ANCOVAs with stress as within‐subject factor, session order as between‐subjects factor and total CTQ score as covariate. To test if cortisol responders would show different connectivity pattern to stress compared with non‐responders (Quaedflieg et al., 2015), we split the participants into ‘responder’ and ‘non‐responder’ groups (R. Miller et al., 2013) and added responder group as a between‐subject factor. In addition, we conducted a separate ANCOVA for the neutral session only, to investigate ELS effects on resting‐state functional connectivity at rest.

### 2.9 Mediation analyses

To test if associations between ELS and stress‐induced changes in resting‐state functional connectivity are mediated by baseline HPA‐axis activity, we carried out three mediation analyses (one for each pairwise connectivity estimate) in the PROCESS macro within SPSS (Version 3.4 by Andrew F. Hayes, http://processmacro.org/index.html). In each model, we regarded CTQ total score as the independent variable *X*, the differential functional connectivity (i.e., the difference between stress and neutral conditions) as dependent variable and basal cortisol (in early afternoon), as a proxy of altered HPA‐axis function, as mediator. Session order was entered as covariate. Associations between CTQ total scores and differential connectivity, without controlling for basal cortisol, were tested as the total effect of the independent variable (*X*) on the dependent variable (*Y*), or Path *c* in each model. Associations of CTQ total scores with basal cortisol were tested as the effect of *X* on the mediator variable (*M*), or Path *a*. Associations of basal cortisol with differential connectivity, controlling for CTQ total scores, were tested as the effect of *M* on *Y*, or Path *b*. Associations between CTQ total scores and differential connectivity while controlling for basal cortisol were tested as the direct effects of *X* on *Y*, or Path *c*′. Finally, the indirect path effect of the association of CTQ total scores and differential connectivity, as mediated by basal cortisol, was tested as the indirect effects of *X* on *Y* through *M* and calculated as the product of Paths *a* and *b*. Statistical significance of the all paths were assessed by bootstrapping (5000 iterations) with 95% CIs.

## 3 RESULTS

### 3.1 CTQ and other questionnaire scores

**FIGURE 2:**
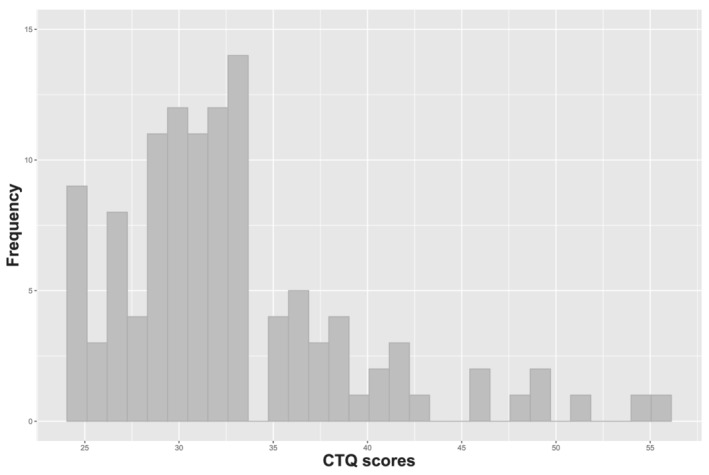
Distribution of CTQ scores. The CTQ scores of 115 participants range from 25 to 56 (M = 32.79, SD = 6.321). CTQ, Childhood Trauma Questionnaire

Descriptive statistics of all questionnaire scores are listed in Table 1. All participants are within normal ranges on all relevant questionnaires (Creamer et al., 1995; Donzuso et al., 2014; Knight, 1984). For instance, BDI scores of all participants were below the cut‐off for moderate to severe depression (i.e., 19 or higher; Beck et al., 1961). Critically, as can be seen in Table 2, the majority of participants had CTQ scores below the criterion for moderate childhood trauma on each subscale of the CTQ, and only approximately 3% of participants had severe scores in one or more subscales across the entire sample. Almost half of participants reported mild exposure to childhood trauma, in particular on the emotional neglect subscale. Thus, the current sample is best characterized as a healthy, highly functioning sample of volunteers with a mild exposure to ELS. CTQ (total) scores (used in analyses below) were positively skewed (see Section 2; Figure 2) and correlated with multiple subscale scores of NEO‐FFI (neuroticism, extraversion and agreeableness), as well as with STAI‐t and BDI scores (see Table 1 for descriptive statistics).

**TABLE 2:** Descriptive statistics of Childhood Trauma Questionnaire subscale scores

	Criteria	Frequency	Mean (SD)
Emotional abuse	None (5–8)	100	6.61 (2.00)
	Low (9–12)	13	
	Moderate (13–15)	2	
	Severe (16+)	0	
Emotional neglect	None (5–9)	62	9.40 (3.37)
	Low (10–14)	46	
	Moderate (15–17)	3	
	Severe (18+)	4	
Physical abuse	None (5–7)	112	5.26 (1.00)
	Low (8–9)	1	
	Moderate (10–12)	1	
	Severe (13+)	1	
Physical neglect	None (5–7)	92	6.41 (1.84)
	Low (8–9)	17	
	Moderate (10–12)	4	
	Severe (13+)	2	
Sexual abuse	None (5)	108	5.11 (.45)
	Low (6–7)	7	
	Moderate (8–12)	0	
	Severe (13+)	0	

### 3.2 ELS alters basal HPA‐axis function

**FIGURE 3:**
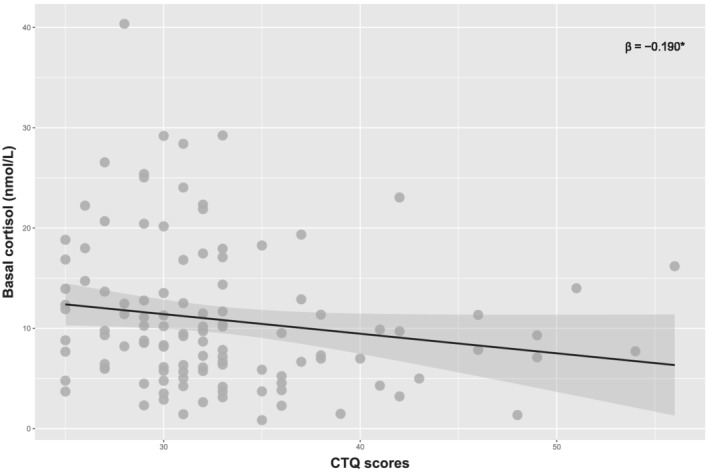
The correlation between basal cortisol (nmol/L) at early afternoon and total CTQ scores across participants. *Bootstrapped 95% confidence interval does not cross zero. CTQ, Childhood Trauma Questionnaire

We first examined if ELS affected baseline HPA‐axis function, using salivary samples taken at home on days without experimental sessions in early and late afternoon. As expected, due to the diurnal cycle of cortisol, baseline cortisol levels in late afternoon were lower than cortisol levels in early afternoon, *t*(114) = 4.15, *p* < .001. We found a negative correlation of CTQ scores with basal cortisol levels in early afternoon, *ρ* (115) = −.227, *p*_*FDR corrected*_ = .03; bootstrapped *β* = −.190, 95% CI: −.402 to −.016 (see Figure 3), but not with basal cortisol in late afternoon, *ρ* (115) = −.016, *p* = .863; bootstrapped *β* = .150, 95% CI: −.202 to .507.

### 3.3 ELS does not affect stress‐induced changes in mood and physiology

**FIGURE 4:**
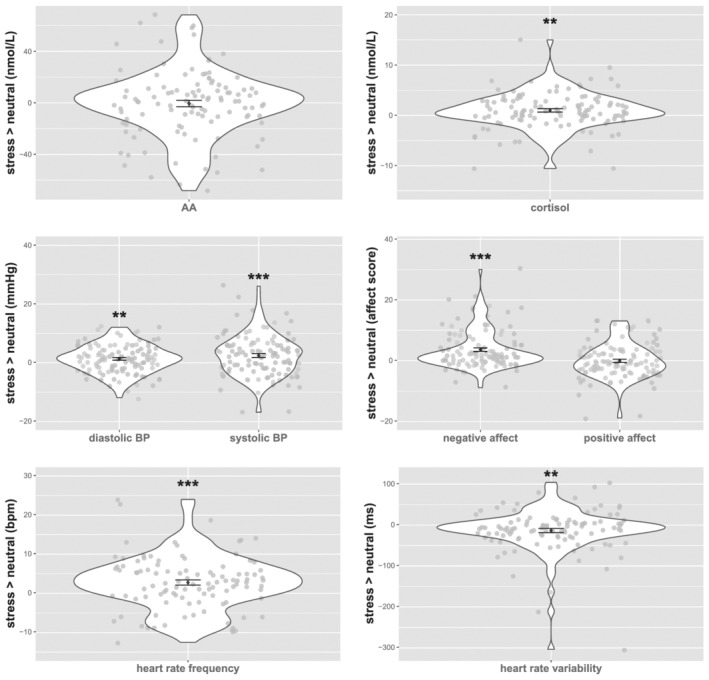
Success of stress induction was verified by different measures at *t* = 20 min, whereas heart‐rate frequency and variability showed differences (stress − neutral) between two sessions during resting‐state scanning (*t* = 49 min − *t* = 56 min). Salivary cortisol, overall BP, self‐reported negative affect and overall heart‐rate frequency were increased due to the stress manipulation, whereas heart‐rate variability was reduced. Error bars represent mean ± SE. ***p < .001; **p < .01; *p < .05. AA, alpha‐amylase; BP, blood pressure

As was reported in an earlier paper on this sample (Henckens et al., 2015), effectiveness of stress induction in experimental sessions was confirmed by several measures (Figure 4). Salivary cortisol (at *t* = 20 min) was elevated in the stress‐induction session compared with the same time point during the neutral session, *F*(1,111) = 9.803, *p* = .002, P*η* ^2^ = .081. Salivary AA (at *t* = 20 min) was not significantly elevated. BP (at *t* = 20 min) was increased relative to the control session, systolic: *F*(1,112) = 15.192, *p* < .001, P*η* ^2^ = .119; diastolic: *F*(1,112) = 8.443, *p* = .004, P*η* ^2^ = .070. Stress induction led to increased negative affect (at *t* = 20 min), *F*(1,112) = 35.732, *p* < .001, P*η* ^2^ = .257, whereas positive affect did not change significantly. HRF during resting‐state scanning (*t* = 49–56 min) was increased during the stress session compared with the neutral session, *F*(102) = 17.238, *p* < .001, P*η* ^2^ = .145, and HRV was decreased, *F*(102) = 9.611, *p* = .003, P*η* ^2^ = .086. All findings survived FDR correction (for all eight tests of effects of acute stress). Within‐session changes in stress measures analysed using repeated‐measures ANOVAs with time as within‐subject factor are reported in the supporting information. We furthermore found no significant main effects of ELS or interactions between ELS and acute stress in any of the physiological and psychological measures. In sum, objective (cortisol, BP, HRF and HRV) and subjective (negative affect) measures confirm successful induction of (mild) acute stress that did not differ between individuals with or without ELS.

### 3.4 ELS affects stress‐induced resting‐state functional connectivity

**FIGURE 5:**
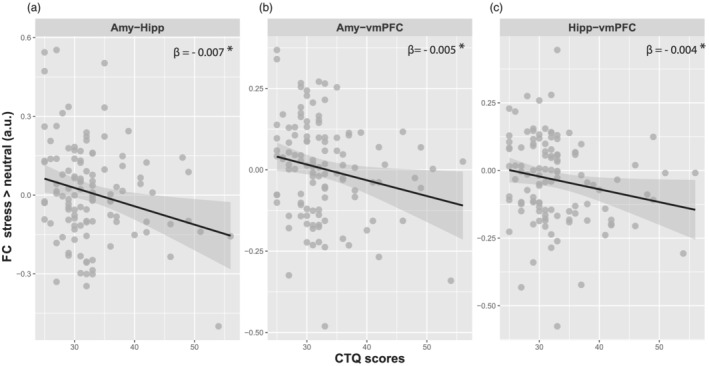
Interactive effects of early‐life stress and acute stress on resting‐state functional connectivity. The three panels show negative correlations between total CTQ scores and stress‐induced changes in amygdala–hippocampal connectivity (a), amygdala–vmPFC connectivity (b) and hippocampus–vmPFC connectivity (c). *Bootstrapped 95% confidence interval does not cross zero. a.u., arbitrary units; CTQ, Childhood Trauma Questionnaire; FC, functional connectivity; vmPFC, ventromedial prefrontal cortex

We next addressed our main hypothesis that ELS alters the effects of acute stress on subsequent resting‐state connectivity within corticolimbic circuits (Figure 5). We computed three pair‐wise functional connectivity measures between amygdala, hippocampus and vmPFC and submitted these to separate ANCOVAs with stress as within‐subject factor, session order as between‐subject factor and CTQ score as covariate. We followed up significant interactions involving CTQ scores with robust bootstrapped regressions (5000 iterations) and report 95% CIs. We found significant interactions of stress and total CTQ scores for amygdala–hippocampal connectivity, *F*(1,112) = 6.649, *p* = .011, P*η* ^2^ = .056; bootstrapped *β* = −.007, 95% CI: −.012 to −.001; *p*_FDR_ = .042, and for vmPFC–amygdala connectivity, *F*(1,112) = 4.616, *p* = .034, P*η* ^2^ = .040; bootstrapped *β* = −.005, 95% CI: −.009 to −.001; *p*_FDR_ = .047. For vmPFC–hippocampal connectivity, the ANCOVA revealed a trend‐level effect, *F*(1,112) = 3.533, *p* = .063, P*η* ^2^ = .031, but the 95% CI of the more robust bootstrapped regression parameter estimates did not cross zero (bootstrapped *β* = −.004, 95% CI: −.009 to .001; *p*_FDR_ = .036). Six follow‐up one‐way ANOVAs were carried out for each connection (three ROIs) and each session (stress and neutral) separately to investigate the directionality of this interaction, but they did not show significant associations with CTQ scores. We also did not observe any main effects of CTQ scores across both sessions.

As amygdala and hippocampus were structurally defined based on individual segmentations of the T1‐weighted structural scans, we were able to also examine effects of ELS on volume of these two bilateral structures. However, Spearman's rank order correlations revealed no association between CTQ scores and these volumetric measures (all *p* > .2).

**FIGURE 6:**
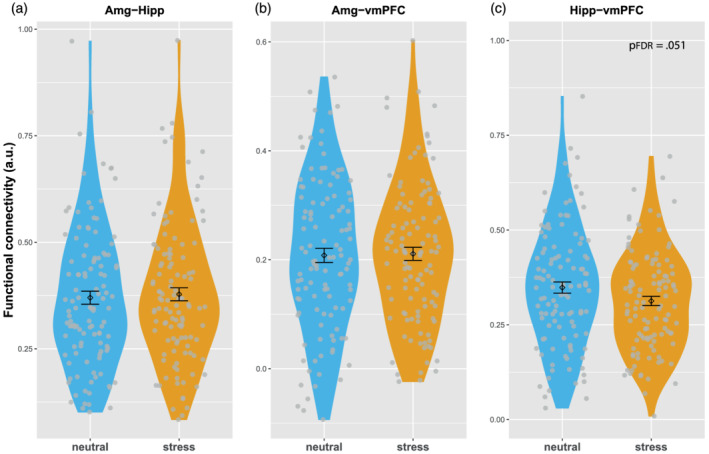
Main effects of acute stress on resting‐state functional connectivity. The three panels show connectivity between amygdala and hippocampus (a), between amygdala and vmPFC (b) and between hippocampus and vmPFC (c). Error bars represent mean ± SE. a.u., arbitrary units; vmPFC, ventromedial prefrontal cortex

We next examined if there were main effects of acute stress on functional connectivity measures (Figure 6). We found that stress reduced vmPFC–hippocampus connectivity at trend level, *F*(1,112) = 5.853, *p* = .017, P*η* ^2^ = .05; *p*_FDR_ = .051, but the other two pair‐wise connectivity measures were not affected by acute stress. To check if effects of acute stress depended on the cortisol response to stress, we divided the participants into cortisol responders and non‐responders (see Section 2) (R. Miller et al., 2013) but found no significant between‐group differences.

In sum, our findings reveal a stronger decrease in functional connectivity in stress versus neutral sessions in individuals with higher CTQ scores.

### 3.5 Basal cortisol negatively mediates the association between ELS and function

To test if basal cortisol levels, as a proxy of altered HPA‐axis function, mediate the observed relationship between ELS and resting‐state functional connectivity change in response to acute stress, we conducted mediation analyses for each of the three ROI pairs. We included session order in our models and used bootstrapped 95% CIs for statistical inference to accommodate the skewness in CTQ scores (see Section 2).

**FIGURE 7:**
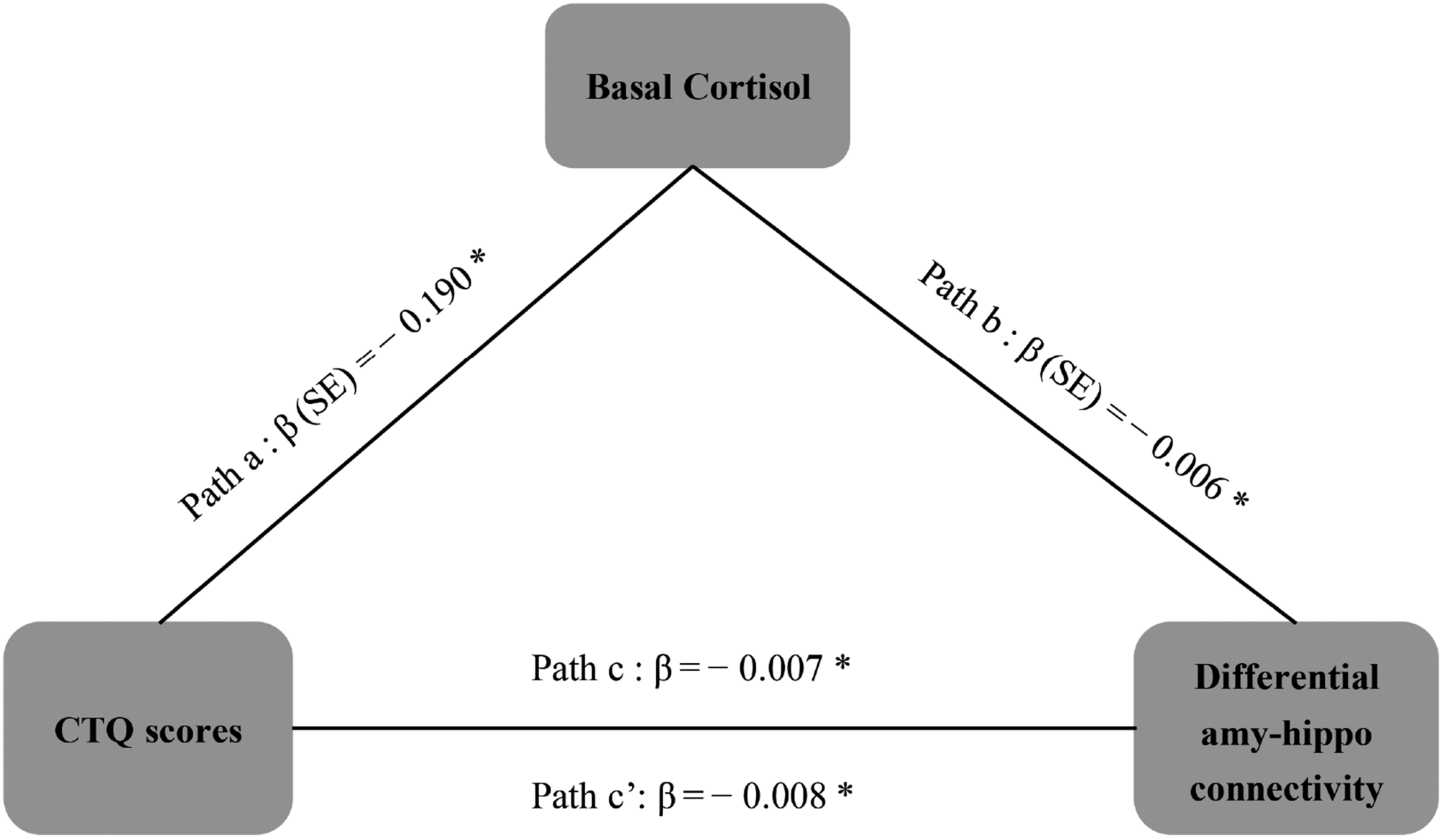
Visualization of mediation model. Path *a* is the association between CTQ scores and basal cortisol. Path *b* is the association between basal cortisol and differential amygdala–hippocampus connectivity. Path *c* is the association between CTQ scores and differential amygdala–hippocampus connectivity. Path *c*′ shows the direct effect of CTQ scores on differential amygdala–hippocampus connectivity. *Bootstrapped 95% confidence interval does not cross zero. CTQ, Childhood Trauma Questionnaire

For all three pathways, we first established (as reported above) that the total effect (Path *c*; see Figure 7) of CTQ scores on differential connectivity is significantly negative (amygdala–hippocampus: *β* = −.0071, 95% CI: −.0127 to −.0012; amygdala–vmPFC: *β* = −.0048, 95% CI: −.0095 to −.0006; hippocampus–vmPFC: *β* = −.0044, 95% CI: −.0088 to −.0005). Also as reported above, higher CTQ scores were associated with lower basal cortisol in early afternoon (i.e., the mediator variable; *β* = −.1904, 95% CI: −.4051 to −.0171; Path *a*; Figure 7). Next, we examined if the mediator variable (baseline cortisol level in early afternoon) is associated with the dependent variables, while including CTQ scores in the model (i.e., Path *b*; Figure 7). This was only the case for differential amygdala–hippocampus connectivity (*β* = −.0057, 95% CI: −.0110 to −.0012). Note that this is a negative association, indicating that the sign of the indirect effect is opposite to the direct effect. For differential amygdala–vmPFC connectivity (*β* = −.0006, 95% CI: −.0046 to .0033) and differential hippocampus–vmPFC connectivity (*β* = .0005, 95% CI: −.0041 to .0045), Path *b* was not significant, and therefore, we did not further test the mediation effect for these pathways.

We then proceeded to examine the direct effect of ELS on differential amygdala–hippocampal connectivity, controlling for baseline cortisol (i.e., Path *c*′; see Figure 7). This association remained significant (*β* = −.0082, 95% CI: −.0139 to −.0027) and even became numerically more negative compared with Path *c*. This suggests that controlling for baseline cortisol made this negative association stronger, rather than reducing or diminishing it, which would be the case in a regular mediation. To allow for statistical inference on this negative mediation effect, we examined the bootstrapped 95% CI of the indirect effect (i.e., the difference between *c* and *c*′; see Figure 7), which was statistically significant: *β* = .0011, CI [.0000 to −.0032].

In conclusion, our mediation analyses reveal a negative mediation effect of basal cortisol levels, as a proxy of altered HPA‐axis function, on the association between ELS and differential amygdala–hippocampal connectivity. Our findings suggest that the negative effect of ELS is buffered in those with lower baseline cortisol levels.

## 4 DISCUSSION

The main aim of this study was to investigate how experience of mild ELS, in group of healthy male adults without history of stress‐related psychopathology, alters the effect of an acute stressor on subsequent brain function. Exposure to ELS has been associated with altered trajectories of brain development, negatively affecting connectivity between corticolimbic circuits involved in emotion processing, including amygdala, hippocampus and (ventromedial parts of) PFC (Bick & Nelson, 2016; Teicher et al., 2016). We therefore specifically focused on acute stress‐related changes in functional connectivity between these regions. Interestingly, the effects of mild ELS on resting‐state functional connectivity between amygdala, hippocampus and vmPFC only became apparent following acute stress. We observed a stress‐induced reduction in functional connectivity between these three regions particularly in those with higher self‐reported ELS. We also found that ELS was associated with lower salivary cortisol levels in early afternoon, indicating reduced basal activity of the HPA axis. Finally, in a mediation analysis, we found that lower basal cortisol statistically *suppressed* the association between ELS and the observed stress‐induced changes in amygdala–hippocampal functional connectivity.

Across participants, we found a (trend‐level) reduction in connectivity between vmPFC and hippocampus following acute stress, but no effect on connectivity of these two regions with the amygdala. Previous studies using stressors consisting of cognitively demanding tasks combined with negative social evaluation (e.g., Trier social stress test or iMAST; Kudielka et al., 2004; Quaedflieg et al., 2013) reported increased post‐stress resting‐state connectivity of the amygdala with hippocampus (Vaisvaser et al., 2013) and vmPFC (Veer et al., 2011; Quaedflieg et al., 2015). Crosstalk between the hippocampus and vmPFC is thought to play a critical role in incorporating novel information into neocortical long‐term memory (Van Kesteren et al., 2010) and transfer of learning to new situations (Gerraty et al., 2014; Zeithamova et al., 2012). Notably, hippocampus and vmPFC are key regions of the default‐mode network (DMN) (Buckner et al., 2008; Raichle et al., 2001), which is implicated in a wide range of internally focused cognitive functions, including self‐referential processing and autobiographical memory (Damoiseaux et al., 2006; Spreng et al., 2009; Spreng & Grady, 2010). By contrast, earlier studies using emotionally arousing audiovisual stressors, such as the one used here, have shown enhanced connectivity between amygdala and regions that are part of the salience network, both during (Hermans et al., 2011) and in the immediate aftermath (Van Marle et al., 2010) of acute stress. The salience network is implicated in arousal, vigilance and attentional reorienting (Hermans et al., 2011). Therefore, our findings may be explained by the nature of the stressor, which may have triggered a prolonged externally focused state of vigilance associated with SN, rather than an internally focused ruminative state, associated with DMN, that is more likely to be triggered by cognitively demanding and evaluative stressors.

We also found that in our sample there was no main effect of ELS on resting‐state functional connectivity when tested across both sessions, or separately in neutral or stress sessions. Also, we found no effect of ELS on amygdala or hippocampal volume. We selected our ROIs based on their critical involvement in emotional regulation and on previous reports of impairments following ELS in terms of structure and function (Herzberg & Gunnar, 2019; Teicher et al., 2016). For instance, ELS has been associated with decreased amygdala–hippocampus, amygdala–(vm)PFC and hippocampus–vmPFC functional connectivity during awake rest (Birn et al., 2014; Burghy et al., 2012; Fan et al., 2014; Kaiser et al., 2018; Ruttle et al., 2013; Sripada et al., 2012), suggesting abnormal trait‐like functional integrity of corticolimbic circuits. Critically, the samples included in those studies varied by onset, type and severity of ELS. Some were healthy individuals who had higher severity of ELS than our sample (Fan et al., 2014), some had experiences of ELS beyond the typically domestic childhood stress reported by our sample (Kaiser et al., 2018; Ruttle et al., 2013) and some were traumatized adolescents who had experienced postnatal parenting stress and financial stress and displayed internalizing symptoms (Burghy et al., 2012) or PTSD symptoms (Birn et al., 2014; Sripada et al., 2012) later in life. Therefore, we think the best explanation for why we did not find main effects of ELS on corticolimbic circuits is that the consequences of ELS as reported by our sample are milder than the sequelae of severe ELS.

The core finding of this study is that there were interactive effects of mild ELS and acute stress on resting‐state functional connectivity (as a proxy for functional integrity of a neural circuit): The effects of mild ELS on functioning of corticolimbic circuitry only became apparent in response to acute stress. To the best of our knowledge, this is the first study to report such an interaction at the neural level, in the population of mildly ELS‐exposed individuals. Developmental theories of stress‐related psychopathology postulate that stress‐related disorders develop through gene‐by‐environment interactions (Carr et al., 2013; Comasco et al., 2015), in which ELS is widely seen as a key environmental factor. ELS, for instance, is strongly associated with development of PTSD upon re‐exposure to trauma (Lanius et al., 2010). Specifically, war veterans who developed PTSD had a higher rate of childhood traumatic events than veterans without PTSD (Bremner et al., 1993; Emery et al., 1991). Our findings suggest that such a vulnerability may be partly conferred, even in a mildly exposed group, through an ELS‐related aberration in corticolimbic systems involved in affective regulation that makes these circuits more sensitive to the effects of acute stress, and thus predispose individuals to develop stress‐related disorders upon stress exposure in later life.

We furthermore found that mild ELS predicted lower basal cortisol levels, but we did not find an association between ELS and the cortisol responses induced by the relatively mild stressor used in our study. A recent meta‐analysis of effects of ELS on HPA‐axis function concluded that, on average, ELS is associated with blunted cortisol responses to stress (Bunea et al., 2017). However, findings of individual studies are mixed, with studies reporting attenuated (Carpenter et al., 2009; Carpenter et al., 2011; G. E. Miller et al., 2007), exaggerated (Pesonen et al., 2010; Tyrka et al., 2008; Vaccarino et al., 2015), as well as not significantly different (Andreotti et al., 2015; Phassouliotis et al., 2013) cortisol responses to stress in individuals with ELS, which may be due to heterogeneity in type and severity of ELS. We did find an association between ELS and lower basal cortisol specifically at early afternoon, when overall levels were higher than during late afternoon. Earlier studies show that indeed the effects of ELS are mostly observed in earlier phases of the diurnal cycle, for example, in the cortisol awakening response, and leading to a flatter diurnal rhythm (G. E. Miller et al., 2007; Yehuda et al., 2001). Previous studies also demonstrated that psychiatric patients with a history of ELS have lower cortisol awakening responses compared with patients without ELS and that this difference in cortisol diminished throughout the day (Hart et al., 1996; Wessa et al., 2006). Individuals with ELS furthermore show stronger suppression of cortisol production by administration of dexamethasone, a GR agonist that activates the negative feedback loop of the HPA axis (Carpenter et al., 2009; Stein et al., 1997). This alteration of GR sensitivity has been suggested to result from a developmental trajectory of initial chronic hyperactivation (Gunnar & Vazquez, 2006; Tarullo & Gunnar, 2006) of the HPA axis before puberty, developing towards to a chronic state of hypoactivation (Fries et al., 2005; Pryce et al., 2005) after puberty due to enhanced negative feedback sensitivity, a process that is understood as an adaptation caused by persistent exposure to excessive cortisol (G. E. Miller et al., 2007). This mechanism was also proposed as predisposing characteristic for development of PTSD (Yehuda et al., 2004). Overall, our observation of reduced basal cortisol in early but not late afternoon in individuals with ELS is therefore consistent with previous findings.

Because of this alteration, we reasoned that altered basal cortisol can serve as a proxy for altered HPA‐axis activity (G. E. Miller et al., 2007; Yehuda et al., 2001), and therefore, we examined using mediation analysis whether lower baseline cortisol could mediate the observed interactive effects of ELS and acute stress on functional connectivity in corticolimbic circuits (Arnsten & Li, 2005; Joëls et al., 2012; Lupien et al., 2007). We indeed found a mediation effect between these three variables. However, this was not a regular mediation effect: Controlling for baseline cortisol did not render the association between ELS and the effect of acute stress on brain connectivity of our ROIs non‐significant or less significant, which would have been expected if baseline cortisol plays a causal role in exaggerating the acute response to stress. Intriguingly, we found a significant *suppression* effect (MacKinnon et al., 2000), showing that the association between ELS and the effect of acute stress became *stronger* after controlling for baseline cortisol. This suggests that the blunted HPA‐axis response following exposure to ELS, which has been described as an adaptation to chronic exposure to stressful environments, also extends to the acute neural response to stress. Indeed, blunted neural responses to acute stress have been observed in people who are at higher risk for developing psychopathology, potentially due to altered baselines occluding effects of acute stress (van Leeuwen et al., 2021). The underlying mechanism of such an effect is complex because, on the one hand, it may involve differences in developmental exposure to stress (hormones), which can lead to structural and functional alterations in the relevant neural circuits as well as changes in (e.g., glucocorticoid) receptor sensitivity (Moriceau et al., 2006; Moriceau et al., 2009; Suderman et al., 2012). On the other hand, it may also reflect immediate consequences of lower circulating glucocorticoids, which are known to interact with catecholamines such as norepinephrine to boost the effects of acute stress (Roozendaal et al., 2009). In sum, although our findings should be interpreted with caution given the low levels of ELS and the fact that we had only single measures of baseline cortisol, our findings warrant more close investigation of the role of blunted glucocorticoid function in development of psychopathology following ELS.

Our study has a number of important limitations. First, we only included male participants. Previous studies have shown that ELS effects depend not only on ELS type, onset and severity but also on sex (Heim & Binder, 2012; Koss & Gunnar, 2018; Strüber et al., 2014; Tarullo & Gunnar, 2006). Because women are also more vulnerable to develop stress‐related disorders (McLean et al., 2011; Olff et al., 2007), it is crucial to extend our findings to females in future research. The reliability of retrospectively recalling ELS in adulthood may furthermore be hampered by inherent biases. In addition, it has been argued that effects of ELS are multidimensional and that a distinction should be made between ELS associated with deprivation (i.e., the absence of expected environmental inputs and complexity) versus threat (i.e., the presence of experiences that represent a threat to one's physical integrity) (McLaughlin et al., 2014). Based on preliminary animal and human research, the deprivation of sensory inputs is thought to prompt synaptic over‐pruning and a subsequent reduction of association cortex. In contrast, chronic threat exposure would lead to chronic activation and adaptation of the HPA axis (McLaughlin et al., 2014) and have lasting effects in corticolimbic circuits examined in the current study. However, in our study, we could not verify this dimensional nature of ELS exposure and thus suggest that this should be investigated systematically in future work. The limitations of using saliva samples taken at home to investigate ELS effects on baseline cortisol should also be mentioned. Although participants received instructions that should minimize effects of confounds on cortisol levels, it was impossible to control whether participants complied with these rules. Therefore, it is possible that cortisol levels were affected by factors such as food consumption and waking time. Finally, it should be noted that the cortisol changes found in response to our stress manipulation were relatively small compared with studies using, for example, the Trier Social Stress Test (van Leeuwen et al., 2021).

In conclusion, our study reveals that a history of mild ELS exacerbates stress‐induced effects on corticolimbic circuits involved in emotional processing, in a sample that is otherwise mentally and physically healthy. Furthermore, our mediation analyses suggest that this effect is buffered in those individuals who exhibit lower baseline levels of cortisol at early afternoon. Thus, our study may reveal part of the mechanism by which adversity experienced during early development may alter stress sensitivity in adulthood.

## CONFLICT OF INTEREST

The authors declare no conflict of interest.

## AUTHOR CONTRIBUTIONS

GF designed the study, HW, JvL, LdV and EH analysed the data and all authors contributed to writing the manuscript.

### PEER REVIEW

The peer review history for this article is available at https://publons.com/publon/10.1111/ejn.15538.

## Supporting information


**Text S1** Outcomes of the repeated‐measures ANOVA on physiological and psychological measures
**Table S1** The effect of stress and its interaction with time and session order on physiological and psychological outcome measures
**Figure S1** Salivary cortisol levels over time. Times are relative to the start of the first movie clip. Asterisks indicate a significant difference between neutral and stress at that timepoint. Error bars represent mean ± SE. ** *p* < 0.01; * *p* < 0.05
**Figure S2** Salivary cortisol levels over time, separated by session order. Times are relative to the start of the first movie clip. There was a significant stress x session order interaction. Error bars represent mean ± SE

## Data Availability

Data will be made available upon reasonable request to the corresponding author.
