# Supplementary material for: Mild early‐life stress exaggerates the impact of acute stress on corticolimbic resting‐state functional connectivity
Source: Eur J Neurosci. 2021 Dec 1;55(9-10):2122–41. doi: 10.1111/ejn.15538 (PMC9299814; doi:10.1111/ejn.15538)
Supplement: Supplementary file 1 — Text S1 Outcomes of the repeated‐measures ANOVA on physiological and psychological measures Table S1 The effect of stress and its interaction with time and session order on physiological and psychological outcome measures Figure S1 Salivary cortisol levels over time. Times are relative to the start of the first movie clip. Asterisks indicate a significant difference between neutral and stress at that timepoint. Error bars represent mean ± SE. ** p < 0.01; * p < 0.05 Figure S2 Salivary cortisol levels over time, separated by session order. Times are relative to the start of the first movie clip. There was a significant stress x session order interaction. Error bars represent mean ± SE [file EJN-55-2122-s001.pdf]

Text S1. Outcomes of the repeated-measures ANOVA on physiological and psychological measures.

We performed a repeated-measure ANOVA with stress and time as within-subject factor and session order as between-subject factor on all physiological and psychological measures. There was a main effect of stress on salivary cortisol [F(1,111=6.555, p = 0.012, Pη2=.056], but no interaction between stress and time. Figure S1 shows that while cortisol levels are higher in the stress condition over all three time points, cortisol levels at t = -15 were higher compared to t = 20 in both conditions [F(1,111)= 7.714, p = 0.006, Pη2=.065)], indicating presumable contamination of stress anticipation. To investigate whether this was in fact the case, we tested the interaction between stress and session order on cortisol levels. Indeed, the stress effect on salivary cortisol differed significantly as a function of session order [F(1,111)=6.159, p = 0.015, Pη2=.053] (see Table S1). Figure S2 shows the directionality of this effect, showing comparable cortisol levels during the first session (independent of condition), but lower starting cortisol during the second session if they had stress during the first session, and higher starting cortisol during the second session if they had neutral during the first session. These findings can be explained by the fact that before taking part in the study, participants were informed that they would go through one stress session and one neutral session, in random order. Together these findings indicate that the first sample is contaminated by stress anticipation. We also investigated the stress x session order interaction for the other outcome measures, find all statistics in Table S1.

Table S1. The effect of stress and its interaction with time and session order on physiological and psychological outcome measures

			F-value	DF	p-value	Effect size (partial eta squared)
Cortisol		Stress	6.555	1, 111	0.012*	0.056
		Stress * Time	0.983	1, 110	0.377	0.018
		Stress * Session order	6.159	1, 111	0.015*	0.053
AA		Stress	0.268	1, 102	0.606	0.003
		Stress * Time	0.451	1, 101	0.638	0.009
		Stress * Session order	0.634	1,101	0.428	0.006
BP	Systolic	Stress	6.719	1,112	0.011*	0.057
		Stress * Time	4.068	1, 111	0.020*	0.068
		Stress * Session order	17.568	1, 112	< 0.001***	0.136
	Diastolic	Stress	3.677	1, 112	0.058	0.032
		Stress * Time	3.281	1, 111	0.041*	0.056
		Stress * Session order	7.589	1, 112	0.007**	0.063
Affect scores	Negative	Stress	59.001	1, 112	< 0.001***	0.345
		Stress * Time	39.168	1, 111	< 0.001***	0.414
		Stress * Session order	5.536	1, 112	0.020*	0.047
	Positive	Stress	0.121	1, 112	0.728	0.001
		Stress * Time	4.049	1, 111	0.020 *	0.068
		Stress * Session order	54.101	1, 112	< 0.001***	0.326

Repeated-measures ANOVAs were used to test the effect of stress and its interaction with time and session order on physiological and psychological outcome measures. These results are visually

presented in Figure S1 and S2. **DF:** degrees of freedom; **AA:** alpha-amylase; **BP:** blood pressure; * $p < 0.05$, ** $p < 0.01$, *** $p < 0.001$

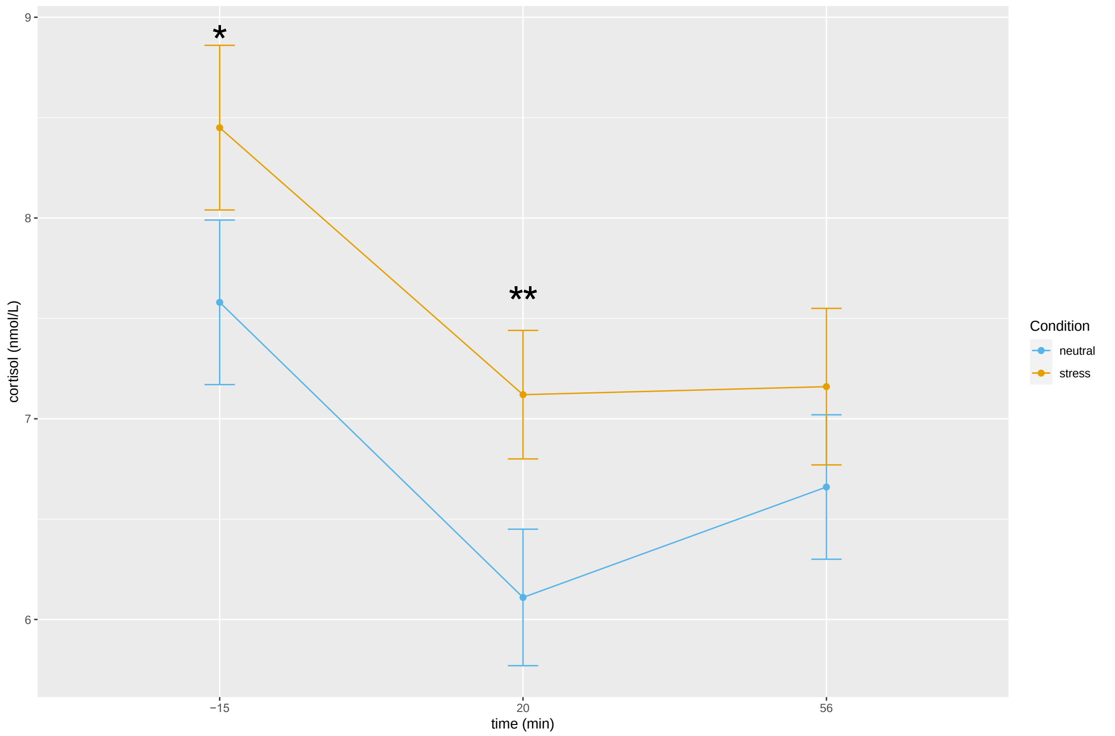

Fig. S1 Salivary cortisol levels over time. Times are relative to the start of the first movie clip. Asterisks indicate a significant difference between neutral and stress at that timepoint. Error bars represent mean ± SE . ** $p < 0.01$; * $p < 0.05$.

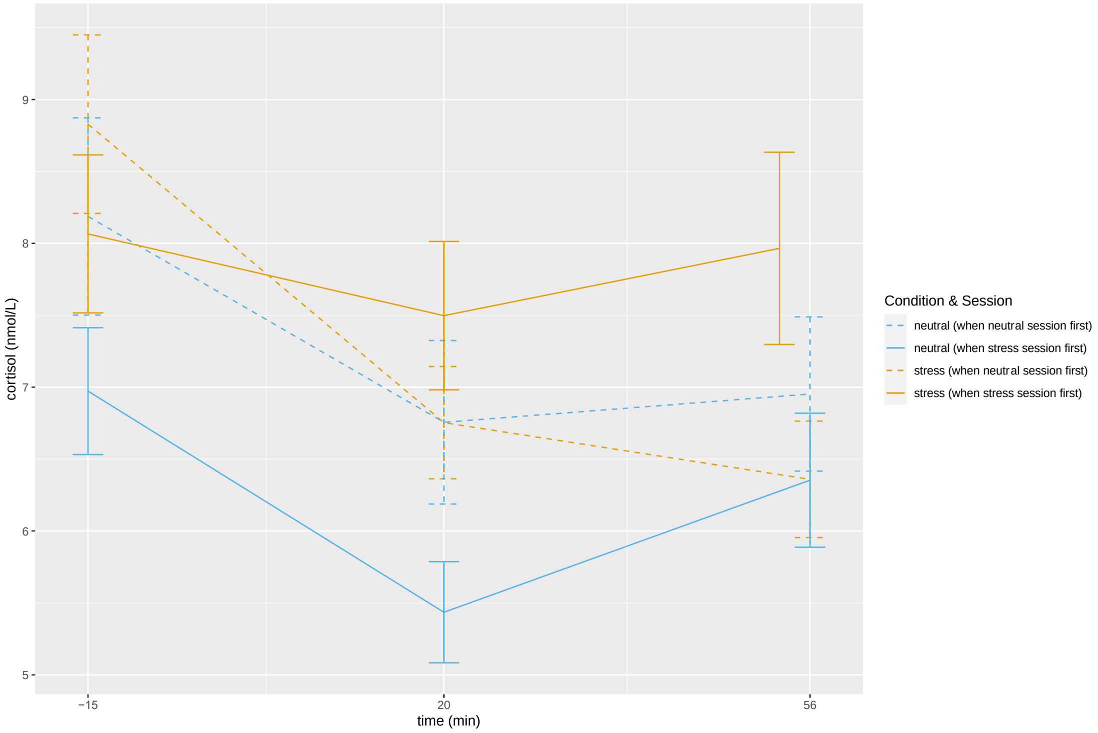

Fig. S2 Salivary cortisol levels over time, separated by session order. Times are relative to the start of the first movie clip. There was a significant stress x session order interaction. Error bars represent mean ± SE.
